# Data set from the Union Army samples to study locational choice and social networks

**DOI:** 10.1016/j.dib.2017.12.007

**Published:** 2017-12-20

**Authors:** Dora L. Costa, Matthew E. Kahn, Christopher Roudiez, Sven Wilson

**Affiliations:** University of California, Los Angeles and NBER, University of Southern California and NBER, University of Maryland, Brigham Young University, United States

**Keywords:** Social networks, Migration, Mortality

## Abstract

We describe the publicly available data created by the NIA funded Early Indicators program project, often referred to as the Union Army data, and the subset of these data used in “Persistent Social Networks: Civil War Veterans Who Fought Together Co-Locate in Later Life” (Costa et al., Forthcoming) [1]. This data subset can be used for reproducibility and extensions and also illustrates how the original complex data derived from archival administrative records can be used.

**Specifications Table**

**Table:** 

Subject Area	Economics
More specific subject area	Social capital
Type of data	STATA
How data was acquired	The data were obtained from archival record collections.
Data format	Raw, partial analyzed and
	Programs to reproduce research paper tables
Experimental factors	Follow-up of random samples
Experimental features	Statistical analysis of location choice
Data source location	United States, c. 1900
Data accessibility	The data are available at http://www.openicpsr.org as Project 100996.
	Underlying data are available at http://www.uadata.org

**Value of the data**

• The data are a rich source of information on the long-term impact of wartime social networks of Union Army veterans on geographic location at both the county and neighborhood level.
• The data permit the study of the long-term impact of wartime social networks on older age mortality.
• The data allow researchers to examine how a pension income transfer and health affect residential choice.
• The data are a reference point for users of the complex data samples which comprise the complete collection

## 1 Data

The Union Army samples are an unparallaled resource for the study of the first cohort of men to reach age 65 in the twentieth century. The samples provide detailed, longitudinal data from youth to death on the health, residence, family structure, pension wealth, and occupation of men who served in the Union Army during the US Civil War (1861–1865). A fortuitous cluster sampling design which led to the collection of entire Civil War military companies make the data ideally suited for the study of social capital and wartime ties. The data, collected from the 1980s to the present, represent a pioneering and still unmatched effort by the Nobel Laureate Robert Fogel to create a longitudinal database for an historical population from administrative records.^1^

The Union Army samples comprise several major collections. The core collection is a sample of 39,338 soldiers, a 1.6% random sample of all whites mustered into the Union Army. A second core collection consists of two random samples of U.S. Colored Troops. Additional collections include over-samples of white Union Army recruits who enlisted in the largest U.S. cities, POWs who survived to 1900, and veterans who lived to 95 years of age or more. In addition, auxiliary data include GIS historical maps and ward characteristics for 6 major US cities.^2^ The data used in “Persistent Social Networks: Civil War Veterans Who Fought Together Co-Locate in Later Life” come from the core sample of 39,338 white soldiers and the urban over-sample of 12,671 recruits. Because the original data come from complex administrative records and from multiple samples, this data set is a useful research tool for researchers not just for replication and extensions but also for understanding how to use the original data samples.

## 2 Experimental design

### 2.1 The core Union Army sample and the urban sample

The core sample of white soldiers and the urban oversample were drawn from white volunteer infantry regiments. The core sample comes from 330 companies, randomly drawn from the complete list of volunteer regiments found in Frederick H. Dyer's *Compendium of the War of the Rebellion* and represents 11% of all infantry regiments. Men who first entered the service as commissioned officers were excluded from the sample to ensure representativeness to the 1860 white, Northern, male population of military age of the United States.

Sampling for the core sample followed a one-stage clustering procedure with companies serving as clusters. Once the companies were randomly selected, names and identifying information were extracted from the Regimental Books (Record Group 94) housed at the National Archives and Records Administration in Washington, DC. Soldiers were then linked to three types of military data set records: military service records, service medical reports, and pension records, including detailed medical reports of examining surgeons.

The collection of the pension data, particularly the reports of the examining surgeons represented one of the big challenges of the project. Nineteenth century medical descriptions in the examining surgeons reports had to be turned into analytical strings with the help of a team of physicians, a programmer, and experienced staff. Inputter training required 6 months and even a trained inputter required one hour to complete the reports of the examining surgeons. A trained inputter required a full hour to input the rest of the pension record.

The urban sample was drawn from a list of infantry companies than had more than half of their recruits enlisting in each of the target cities.^3^ A total of 94 companies were randomly selected in proportion to the 1860 population of the target cities. Thus the numbers of companies consists of 37 from Manhattan and Brooklyn, 20 from Philadelphia, 12 from Boston, 13 from Chicago, and 12 from Baltimore. Linkage to military records then proceeded as for the core Union Army sample. The final sample consists of 12,624 soldiers, 52% of whom can be linked to any one of the five cities at some point in their lives. The remainder cannot either because they never lived in the city (though they might have enlisted there) or they died during the war.

Both samples were linked to pre- and post-war manuscript census schedules to obtain additional demographic, geographic, family, and socioeconomic information. The Union Arm sample was linked to the 1850, 1860, 1900, and 1910 records. Advances in online resources (including the creation of indices) and the release of additional census years led to the linkage of the urban sample to every decade between 1850 and 1930, with the exception of the burnt 1890 census. Because of funding limitations, the original Union Army sample was never linked to these additional censuses. Linkage was done by hand by trained inputters and genealogists to maximize linkage rates and reduce false positive rates.

Table 1, which presents sample means for selected variables, shows, unsurprisingly, that men in the urban sample were more likely to be shorter, foreign-born, and laborers compared to a random sample of all soldiers. They were less likely to die within the service but were less likely to be known to be alive in 1900, consistent with the higher mortality rates faced by urban compared to rural residents. They also were less likely to ever show up on the pension rolls, a finding consistent with their higher mortality rates. Because the pension program was liberalized only in 1890, city dwellers were more likely to die before making it to the rolls. Conditional on being observed alive in 1900, over 80% of veterans were linked to the 1900 census.^4^

**Table 1:** Mean characteristics of the complete Union Army sample and the urban oversample.

	Union Army	Urban
Characteristics at enlistment		
Year of enlistment	1862.655	1862.194
Age	25.726	26.240
Height (inches)	67.645	66.837
Foreign-born	0.254	0.498
Occupation (fraction) is		
Farmer	0.497	0.126
Professional/proprietor	0.051	0.092
Artisan	0.164	0.243
Laborer	0.240	0.374
Fraction who died in the war	0.149	0.129
Fraction of war survivors who ever received a pension	0.596	0.381
Fraction of war survivors known to be alive in 1900	0.440	0.381
Fraction of all enlistees known to be alive in 1900	0.374	0.225
Fraction of all enlistees known to be alive in 1900		
who are linked to the 1900 census	0.837	0.801

**Fig. 1:**
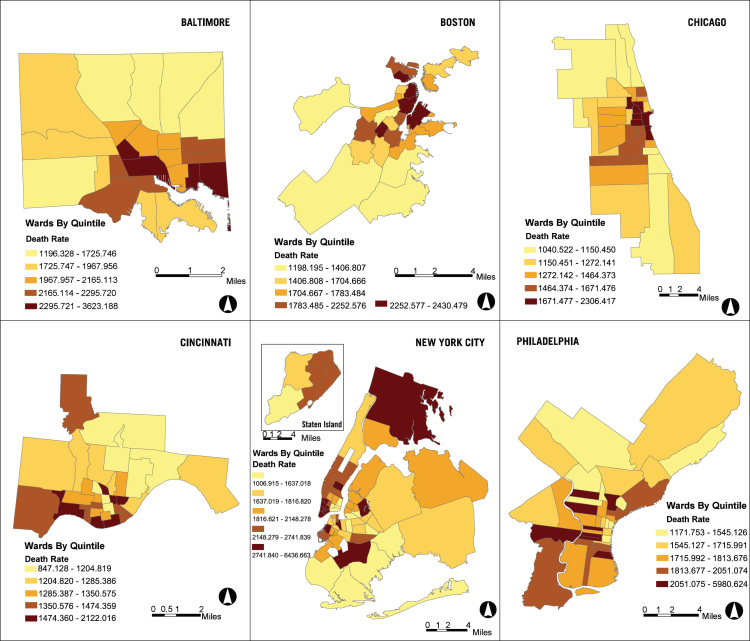
Death rates per 100,000 within city wards in 1900.

**Fig. 2:**
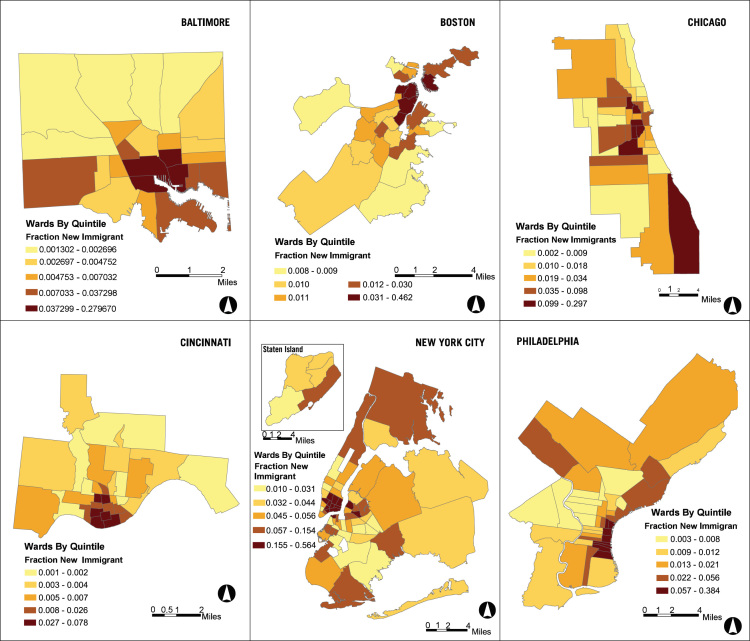
Fraction of new immigrants within city wards in 1900.

A companion collection to the urban sample is the Historical Urban Ecological (HUE) data set for seven of the largest, Northern cities – Baltimore, Boston, Brooklyn, Chicago, Cincinnati, Manhattan, and Philadelphia from 1830 to 1930. The three main components of the database are historical GIS ward boundaries, historical GIS street networks from circa 1930, and ward-level data from annual city reports. Fig. 1 shows death rates per 100,000 by ward for Baltimore, Boston, Chicago, Cincinnati, New York City (all boroughs), and Philadelphia in 1900. Fig. 2 illustrates where new immigrants were located using data from HUE and from the complete count census indices available from the Minnesota Population Center and Ancestry (2013).

### 2.2 The analytical samples

Our three analytical samples consist of 1) all uninstitutionalized veterans from the core Union Army sample for whom we know county of enlistment and county of residence in 1900 and for which we have information on county characteristics in 1900 from published census data; 2) all uninstitutionalized veterans, from either the core Union Army sample or the urban sample, whom we could place in a ward in 1900 in Baltimore, Boston, Chicago, Cincinnati, New York City (all boroughs, including Brooklyn), and Philadelphia and for whom we know city of enlistment; and 3) the same sample as in 2), restricted to veterans for whom we know age at death. Details on the construction of the analytical samples are provided in “Persistent Social Networks: Civil War Veterans Who Fought Together Co-Locate in Later Life.”

Twenty-seven percent of veterans in the county sample remained in their county of enlistment (see Table 2 which presents sample means for individuals, chosen counties, and all US counties). Their 1900 county of residence was on average 244 miles from their enlistment county. They avoided the former Confederacy and lived in counties with fellow veterans. They were more likely to be farmers at enlistment than the Army as a whole, both because mortality rates were lower in rural areas and because rural residents are easier to link to the census. Only 6% of veterans in the county sample are in the counties of the six major cities in the ward sample.

**Table 2:** Mean characteristics of county locational choice sample.

Mean Characteristics of:	Veterans	Chosencounties	Allcounties
Enlistment occupation (fraction) is			
Farmer	0.582		
Professional/proprietor	0.068		
Artisan	0.181		
Laborer	0.158		
Fraction remaining in county of enlistment	0.266		
Monthly pension amount in 1900 ($)	9.989		
Fraction in poor health	0.235		
Distance from enlistment county (miles)		244.038	811.578
Population		138,142	26,720
Mean February temperature (Fahrenheit)		29.280	36.183
Dummy=1 if coastal county		0.153	0.104
Dummy=1 if ex-Confederate county		0.021	0.368
Fraction of new immigrants per population		0.016	0.009
Fraction of population in manufacturing		0.066	0.030
Percentage voting for McClellan in 1864		42.969	15.604
Percentage voting for McKinley in 1900		55.416	39.921
Number of veterans from the same company		4.403	0.011
Number of veterans		13.978	2.448
Number of veterans from the same prewar town		0.705	0.001
Number of veterans from the same birth city		0.515	0.001

Half of all veterans in the ward sample of six major cities had remained in their city of enlistment (see Table 3 which gives sample means for individuals, chosen wards, and all wards of the six major cities). Their city of enlistment was on average 120 km from their residence in 1900. Veterans were more likely to live in wards with other veterans, were concentrated in New York City and Philadelphia, and lived further away from the central business district.

**Table 3:** Mean characteristics of ward locational choice sample.

Mean Characteristics of:	Veterans	Chosenwards	Allwards
Enlistment occupation (fraction) is			
Farmer	0.035		
Professional/proprietor	0.221		
Artisan	0.375		
Laborer	0.309		
Fraction remaining in city of enlistment	0.500		
Fraction in 1900 in			
Baltimore	13.24		
Boston	7.21		
Chicago	18.31		
Cincinnati	3.89		
New York City	30.28		
Philadelphia	26.96		
Monthly pension amount in 1900 ($)	7.298		
Fraction in poor health	0.296		
Irish-born	0.103		
German-born	0.178		
City population in 1900		1,821,738	1,692,285
City death rate per 100,000		4658.146	4274.176
Distance of city from city of enlistment (in km)		120.124	514.090
Ward population		72,320	35,136
Ward population density		0.017	0.019
Ward death rate per 100,000		1846.985	1863.89
Distance to city center (in meters)		18,380.65	10,097.064
Fraction of new immigrants per population		0.039	0.062
Fraction of Irish-born per population		0.064	0.068
Fraction of German-born per population		0.077	0.076
Fraction of blacks		0.042	0.045
Number of veterans from the same company		0.551	0.038
Number of veterans		7.978	3.838
Number of veterans from the same birth city		1.151	0.152

Among the veterans in the ward sample for whom we know cause of death, the average number of years lived after 1900 was 14 years (see Table 4 for sample means). Given that average age at baseline was 60, men thus died at roughly age 74. Averaging over all years lived, veterans could expect to have in the same ward less than one man from the same company, almost 4 other veterans, and less than one veteran from the same birth city but not from the same company. Seventy-four percent of the time a veteran could expect to have a wife.

**Table 4:** Mean characteristics of mortality sample.

	Mean
Months lived after 1900	165.213
Time-varying characteristics:	
Number of veterans from the same company in the same ward	0.485
Number of veterans in the same ward	3.813
Number of veterans from the same birth city but not the same company in the same ward	0.409
Dummy=1 if wife is alive	0.737
Baseline characteristics:	
Age in 1900	60.474
Ward death rate per 100,000 in 1900	1835.35
Dummy=1 if	
in poor health	0.323
wounded in war	0.352
enlisted in city of 50,000+	0.648
Irish-born	0.095
laborer at enlistment	0.305
out of the labor force in 1900	0.272
laborer in 1900	0.341
in 1900 living in same city of enlistment	0.498

## 3 Conclusion

The Union Army samples were originally collected to study the determinants of later life work levels, disease, and death for the early twentieth century and provide one of the few sources of detailed health information for a past population. The fortuitous cluster sample design has made the data a rich source of information for the study of the impact of social networks on wartime loyalty and post-war migration. The Union Army samples remain an untapped source for the study of fertility, nuptiality, and the effective delivery of government benefits.

Under NIA grant P01 AG10120, Early Indicators, Intergenerational Processes and Aging (Dora Costa, PI), we have been collecting data on the children of Union Army soldiers who survived to 1900 to study the intergenerational transmission of parental stress on child mortality and socioeconomic status. The samples that are being created consist of an over-sample of the children of white POWs, a random sample of the children of white soldiers, and a random sample of the children of black soldiers. Both daughters and sons are being linked to all of the censuses from 1850 to 1940 (with the exception of 1890 which was destroyed in a fire) and to mortality records. Because of the detailed data available in the pension records and on-line, particularly for white soldiers, the resulting sample promises to be one of the few historical samples with equally rich information on daughters as well as on sons.

## Funding sources

Dora Costa, Christopher Roudiez, and Sven Wilson gratefully acknowledge the support of NIH Grant P01 AG10120. Dora Costa also acknowledges the use of facilities and resources at the California Center for Population Research, UCLA, which is supported in part by NICDH Grant P2C R24HD041022.

## Footnotes

1 The data were largely collected since 1991 under NIA grant P01 AG10120, Early Indicators of Later Work Levels, Disease and Death (Robert Fogel, PI) and have continued to be cleaned and coded under NIA grant P01 AG10120, Early Indicators, Intergenerational Processes and Aging (Dora Costa, PI).

2 See uadata.org for data downloads and detailed descriptions. The GIS and ward data also are available as ICPSR 35617.

3 The list was drawn from Dyer's *Compendium*, State Adjutant Generals reports, and various on-line resources.

4 Modern technologies would have produced higher linkage rates in the original Union Army sample. Linkage rates should be lower in the urban sample because large city dwellers are always more difficult to locate.
