# Supplementary material for: Data set from the Union Army samples to study locational choice and social networks
Source: Data Brief. 2017 Dec 20;17:226–33. doi: 10.1016/j.dib.2017.12.007 (PMC5988018; doi:10.1016/j.dib.2017.12.007)
Supplement: Supplementary file 1 — Supplementary material [file mmc1.docx]

Conflicts of Interest: None
